# Supplementary material for: Chromothripsis during telomere crisis is independent of NHEJ, and consistent with a replicative origin
Source: Genome Res. 2019 May;29(5):737–49. doi: 10.1101/gr.240705.118 (PMC6499312; doi:10.1101/gr.240705.118)
Supplement: Supplemental Material [file supp_gr.240705.118_Supplemental_file_1.zip › contigs/annotated_contigs/DB78/contig.2.DB78_length_557_mean_cov_12.3859964093.docx]

**DB78_length_557_mean_cov_12.3859964093**

```
ATCCTTATTTTTCCTAAAATGAAGCAAAACAAACCAAACTGAAACCAATTCACAAAGAAAGACAACTCCCATACCAGTATAACTATTAT
 >chr5:93536413-93536673 + E=8e-145 p=0e+00
CTCCTACTCCTACTCCTTAAATTTCTGCTCCCTTTTATGTTGACGCTCCTTTTTCTTGATTTCTTTTTTCCTGCTATATTCCTCACTTA

GAGGACACAGGTCCATTGTTCCTCAAAGCTGCTATTTCTCTAGAAATATAGAATTTAAAC|ATATATATATATATATATATAT|TTTGT
                                                             >chr5:93540983-93541302 +
TTTGTTTTGTTTTTGAGACGGAGTCTTGCTCTGTCACCCAGGATGGAGTGTTGTGGCGTGATCTCGGCTCACTGCAACCTCCGCCTCCT
E=2e-177
GGGTTCATGCCATTCTCCTGCCTCAGCCTCCCGAGTAGCTGGGACTACAGGTGCCCACCACCACTTTTTTATATTTTTAGTAGAGATGG

GGTTTCACCATGTTAGCCAGGATGGTCTCGATCTCCTGACCTCGTGATCCGCCCGCCTCGGCCTCCCAAAGTGCTGGGATTACAGGCGT

GAGCCACCATGCCCGGCCCAATCTT
```
